# A Brief History in Cardiac Regeneration, and How the Extra Cellular Matrix May Turn the Tide

**DOI:** 10.3389/fcvm.2021.682342

**Published:** 2021-05-20

**Authors:** Atze van der Pol, Carlijn V. C. Bouten

**Affiliations:** Eindhoven University of Technology, Department of Biomedical Engineering, Eindhoven, Netherlands

**Keywords:** cardiac regeneration, extra cellular matrix, stem cells, heart failure, developmental biology

## Abstract

Tissue homeostasis is perturbed by stressful events, which can lead to organ dysfunction and failure. This is particularly true for the heart, where injury resulting from myocardial infarction or ischemic heart disease can result in a cascading event ultimately ending with the loss of functional myocardial tissue and heart failure. To help reverse this loss of healthy contractile tissue, researchers have spent decades in the hopes of characterizing a cell source capable of regenerating the injured heart. Unfortunately, these strategies have proven to be ineffective. With the goal of truly understanding cardiac regeneration, researchers have focused on the innate regenerative abilities of zebrafish and neonatal mammals. This has led to the realization that although cells play an important role in the repair of the diseased myocardium, inducing cardiac regeneration may instead lie in the composition of the extra cellular milieu, specifically the extra cellular matrix. In this review we will briefly summarize the current knowledge regarding cell sources used for cardiac regenerative approaches, since these have been extensively reviewed elsewhere. More importantly, by revisiting innate cardiac regeneration observed in zebrafish and neonatal mammals, we will stress the importance the extra cellular matrix has on reactivating this potential in the adult myocardium. Finally, we will address how we can harness the ability of the extra cellular matrix to guide cardiac repair thereby setting the stage of next generation regenerative strategies.

## Introduction

Heart failure (HF) is a leading public and clinical health problem of the 21st century with a prevalence of more than 23 million worldwide (1, 2). HF as a result of myocardial infarction (MI) and ischemic heart disease remains the most prominent health challenge of the developed world, with a 5 year survival rate of <50% (3). The pathophysiology of HF resulting from MI or ischemic heart disease is characterized by the acute or gradual loss of functional cardiomyocytes. The remaining cardiomyocytes ineffectively attempt to compensate for the loss of myocardium, initiating a cascade of biological processes which eventually lead to cardiac remodeling. Cardiac remodeling induces scar tissue formation, ventricular wall thickening, and eventually diminished cardiac muscle functionality. Current clinical therapeutic interventions allow to slow down the progression of post-infarction HF, but are unable to cure the loss of functional tissue, i.e., to replace myocardial scaring with functioning contractile tissue. Such therapies include several forms of drug treatments (e.g., ACE inhibitors), lifestyle modifications, surgery and ultimately heart transplantation. Cardiac transplantation is to date the only curative option for end-stage heart failure but due to the low number of organ donors and the side effects of immunosuppressive drugs, only a few thousand patients a year have access to a transplantation program. Making it increasingly urgent to devise novel approaches to treat these patients.

Over the past decade multiple advances have been made in our understanding of myocardial cell homeostasis, stem cell biology, and matrix biology facilitating the identification of several novel therapeutic strategies directed at healing the myocardium. Many regenerative strategies have focused on cell-based approaches, however the heart is more than just the sum of its parts. The cellular composition of the heart plays a large role in the onset/development of post-infarction HF, however an equally great part is played by the micro-environment of the cell (i.e., the ECM and the cell-matrix interactions) when looking at cardiac repair, remodeling, and injury. The presence of aberrant ECM, as is the case in post-infarction HF development/progression, can reduce or even block the effectiveness of cell-based therapies to repair the heart (4). Interestingly, clues have emerged that the ECM is involved in development and in tissue repair in various organisms (e.g., zebrafish), not just for the heart but also within other organs (5). This begs the question: what would happen if the innate cardiac cells in the injured myocardium are provided with a healthy matrix/micro-environment?

With an eye to the future, in this review we will briefly revisit the shortcomings of stem cell-based approaches and stress how understanding the innate ability of the ECM in zebrafish and neonatal mammals for cardiac regeneration may offer alternative approaches to advance curative therapeutic strategies for patients suffering from post-infarction HF.

## Stem-cell Based Cardiac Regeneration: A Futile History

With the goal of being a one-shot solution to cardiac injury, researchers have attempted to identify a cell source capable of inducing cardiac tissue regeneration. This has led to the investigation of several putative stem-cell based therapies to either stimulate the endogenous cells or deliver exogenous stem cells to trigger cardiac regeneration in the preclinical animal setting and in clinical trials; including embryonic stem cells (ESCs), induced pluripotent stem cells (iPSCs), bone marrow-derived stem cells, and most recently cardiac stem cells (CSCs). Despite these immense efforts, only limited and temporary clinical benefits have been observed, and whatever effects have been largely explained by paracrine cues associated with aiding the reduction of the inflammatory response (6, 7).

Of all the stem cell-based approaches, bone marrow-derived stem cells have been the most extensively studied for their cardiac regenerative potential, and over the years a multitude of experimental and clinical studies have been performed. A recent meta-analysis of 43 randomized controlled clinical trials, with 2,635 patients, focused on exploring the results at 6 months, 1 year, 3 years, and 5 years following the exogenously administration of bone marrow stem cells in patients with acute myocardial infarction (8). The authors observed a minor, but significant improvement, in left ventricular ejection fraction (LVEF) at 1 year, when compared to the control groups. However, at the 3 and 5 year follow-up, there was no significant difference in LVEF between the cell therapy and control groups, suggesting a severe decline in cardiac function in treatment group. A similar trend was observed when the authors looked at infarct sizes. These temporary improvements observed with bone marrow-derived stem cells have also been made in the few studies that have administered human ESC in to HF patients, although the long term effects of this approach have yet to be explored (9, 10). One important pitfall within these studies utilizing bone marrow-derived stem cells or ESC is that none of these studies demonstrated correct integration of the cells within the injured myocardium. Like ESCs, iPSCs derived cardiomyocytes have been recently shown to possess the ability to contract, form gap junctions, and even express the receptors essential for hormonal regulation of the myocardium (11, 12). These cells have shown great promise in the experimental setting, however the risk of vector and/or pluripotency-associated teratoma induced immunogenicity has to be circumvented prior to the initiation of cell based human clinical trials (13). Additionally, as with the previous studies utilizing bone marrow-derived stem cells or ESC, it is highly unlikely that a pure cell-based strategy with iPSC will result in long term beneficially effects. This may lie in the fact that these cells are unable to survive in the injured myocardium micro-environment, and therefore long term beneficial effects may not be achievable with this approach.

The final, and most controversial target for cardiac stem cell-based regeneration are the endogenous CSC. The notion that the adult heart is not just a post-mitotic organ, but has a limited extend for self-renewal, came about from advances in radiocarbon dating (14). In addition several populations of CSC residing within the heart have been identified (4). It has been suggested that these CSCs are responsible for reconstituting a finite basal rate of cardiomyocyte loss, although a direct link between cardiomyocyte renewal and these CSCs has yet to be demonstrated (15). These early findings have pointed to possibilities of utilizing resident cardiac progenitor populations for cardiac regeneration, however recent findings have brought into question the existence of these CPC in the heart (16–18). It has become evident that the renewal of cardiomyocytes in the adult myocardium is mainly due to the proliferation of existing cardiomyocytes rather than by the differentiation of putative progenitor cells (16, 19). Additionally, several recent lineage tracing studies have found that c-kit cardiac progenitors capacity to differentiate into cardiomyocytes is only 0.002–0.035% (16). These recent observations bring severe doubt into the regenerative potential of CPC, making it clear that alternative approaches must be developed to achieve post-injury myocardial regeneration.

Based on these significant advances in stem cell biology, it has become apparent that although healthy stem cells with cardiomyogenic potential are important, they can by themselves not lead to the long term goal of repairing the injured myocardium. This is in large part due to the toxic effects the pathological micro-environment, specifically the diseased extra-cellular matrix (ECM), has on the regenerative capacity of the myocardium. A recent paradigm shift has suggested that the albeit limit beneficial effect of cell-based approaches may not lie in cell restoration, but may in fact lie in their paracrine actions and more importantly ECM homeostasis (20). This ECM homeostasis is achieved by the stem cell-related production of ECM components (collagens, matrix metalloproteinases, tissue-derived inhibitors, anti-inflammatory/immunosuppressive factors) and by the consumption of pro-apoptotic and inflammatory molecules (20). Therefore, it is becoming increasingly evident that the key to push the field of cardiac regenerative medicine forward may not lie on the cellular level alone, but may instead lie in the composition of the ECM and the interaction of cardiac cells with the ECM. Important clues related to the role of the ECM in driving cardiac regeneration have been made while characterizing the innate ability of the zebrafish and neonatal mammalian hearts for self-repair.

## Lessons Learned from Innate Cardiac Regeneration: A Brief History

Within the animal kingdom there are numerous examples of spontaneous organ regeneration following injury. In terms of cardiac regeneration, it is the zebrafish that stands out the most. Zebrafish possess the ability to undergo complete cardiac regeneration without scar formation after the resection of up to 20% of the ventricular apex. Complete re-growth of the amputated region, including the coronary vasculature, myocardium and endothelium is achieved at 60 days post-resection, resulting in a fully functional heart (21–23). This model for cardiac regeneration has been useful in unraveling certain aspects of the regenerative mechanisms, but since it is based on the removal of heart tissue rather than its damage, it is not the most accurate model to study human cardiac damage and repair. To this end a recent study has characterized the cellular response and the ability for functional repair (i.e., regenerative capacity) of the zebrafish heart after cryoinjury, a procedure that, unlike resection, more closely models the pathophysiological process undergone by the human heart (24). Damage was induced to 25% of the ventricle by means of cryocauterization, resulting in massive cardiomyocyte cell death within the injured area and the near coronary vasculature. Cell death in turn induced a proliferative response in the endocardium, epicardium, and myocardium, ultimately resulting in the formation of a scar at the site of injury. However, unlike in the injured human heart, the fibrotic scar tissue was degraded and replaced by functional cardiac tissue, suggesting that myocardial regeneration can occur even in the presence of scar tissue (25, 26). Albeit the change of tissue composition in terms of matrix and cellular components was not fully characterized, these results indicate that there are certain endogenous mechanisms involved with scar tissue regression and cardiac tissue replacement. The characterization of these mechanisms underlying heart regeneration in such animals may offer a way to identify novel strategies to overcome the limited regenerative response in mammals.

The mammalian heart has always been considered to be a post-mitotic organ without the capacity for self-renewal, where growth is achieved through cellular hypertrophy. Recently, however, several studies have identified that cardiomyocytes retain the capacity to proliferate to a limited extend in the postnatal heart of both mice, rats, pigs, and humans (14, 27–31). Research has also shown that the immature mammalian heart possess a greater regenerative potential than previously expected (14). Given the similarities between the adult zebrafish heart and the immature mammalian heart, Porrello *et al* induced cardiac damage, by resection of 15% of the ventricular apex, in 1-day-old neonatal mice (32, 33). Surprisingly, the apex was progressively regenerated, with full restoration of the resected myocardium within 21 days. This regenerative ability of the immature heart was lost once the mice became over seven days old. More recently, similar observations have been made in rats and pigs (29, 30). And even more fascinating several case reports have demonstrated a similar phenomenon in newborn human babies (34, 35). This suggests that at birth mammals retain a surprising cardiac regenerative ability, which is lost upon maturation. Understanding which intrinsic mechanisms are involved in neonatal mammalian cardiac regeneration has triggered a new field of research into characterizing this innate ability for cardiac repair, which may facilitate the identification of novel strategies for cardiac regeneration in the failing heart.

## The ECM in Cardiac Regeneration: An Emerging History

The regenerative potential of zebrafish and of neonatal mammals is truly astonishing, and to this day still baffles the scientific community. In spite of this, recent advances in stem cell biology, molecular biology, and matrix biology have started to unravel this mystery and suggested a key role in cardiac regeneration is played by the constitution of the ECM (36, 37). One of the earliest studies to focus on the regenerative potential of the ECM, utilized decellularized zebrafish cardiac ECM and injected this ECM solution into a murine MI model (38). Surprisingly, this study found that zebrafish ECM was able to stimulate the repair of the diseased myocardium, by inducing cardiomyocyte proliferation, reducing infarct size, and thereby overall improving cardiac function. In an *in vitro* setting the zebrafish ECM was also found to have a positive effect on the proliferation of human cardiac progenitor cells. A similar observation was made utilizing fetal, neonatal and adult cardiac ECM from rats to assess the potential of this ECM for neonatal rat cardiomyocyte expansion *in vitro* (39). The authors found that fetal cardiac ECM improved the overall adhesion and resulted in a 4-fold increase in cardiomyocytes population, when compared to adult cardiac ECM. Analogous observations were made when utilizing fetal pig ECM in a murine MI model (40). Since cross-species strategies might limit the translational potential of this approach, as is the case with injecting zebrafish ECM within a murine MI model, a recent study has also assessed the regenerative potential of the neonatal murine ECM (41). In this study decellularized neonatal cardiac ECM from mice was injected into a murine MI model. As with to the early zebrafish studies it was observed that the neonatal ECM limited scar tissue formation in the left ventricle and promoted revascularization of the injured region. These observations suggest that the fetal/neonatal cardiac ECM has a profound impact on the regenerative potential of the myocardium, and might explain the regenerative capacity of the fetal/neonatal mammalian heart, albeit the role of the individual and combined ECM components has not yet been fully analyzed (14, 27–30). The ECM is a complex mix of various components and to further explore this approach for clinical application it is essential to understand the exact composition of the ECM and which components are responsible for the regenerative potential of the fetal/neonatal ECM.

Advances in RNA sequencing and proteomics analysis, have enabled researchers to begin to unravel the components of the cardiac ECM which are responsible for its regenerative potential (41–49) (Table 1). Utilizing zebrafish as a model for cardiac regeneration, several studies have characterized the composition of the regenerative zebrafish ECM (42, 43). These studies identified that the regenerating cardiac zebrafish ECM had an abundance in hyaluronic acid (HA), an unbranched glycosaminoglycan polymer widely distributed in the ECM. Interestingly, depending on the molecular weight of HA, it has different biological activities such as promoting angiogenesis, inhibiting cell adhesion, and suppressing fibrotic tissue formation (56). The pathway for the production of HA is highly enriched within injured zebrafish myocardium, suggesting an important role in the regeneration of the heart (43). Indeed, when HA production is inhibited, this results in a total block of cardiac regeneration in zebrafish (43). A similar study also identified the ECM component fibronectin being highly expressed in the regenerating zebrafish heart (48). Interestingly, when inducing a loss-of-function mutation of fibronectin in zebrafish, this significantly reduced their cardiac regenerative abilities (48).

**Table 1:** Differential Expression of extracellular matrix (ECM) components in regenerative vs. non-regenerative hearts in different species.

	**Regenerative**			**Non-regenerative**		**Studies demonstrating ECM**
**ECM**	**Zebrafish**	**Neonatal**	**Neonatal**	**Adult**	**Adult**	**component involvement in**
**component**		**rat**	**mice**	**rat**	**mice**	**cardiac regenerative**
Agrin			**↑** (46)		**↓** (46)	Recombinant Agrin promotes cardiac regeneration (45, 46)
Collagen-I	**↑** (50)	**↓** (39)	**↓** (36)	**↑** (39)	**↑** (36)	
Collagen-III		**↓** (39)	**↓** (36)	**↑** (39)	**↑** (36)	
Collagen-IV	**↑** (42)	**↑** (39)		**↓** (39)		
Collagen-V	**↑** (42)	**↓** (39)		**↑** (39)		
Collagen-VI		**↑** (39)		**↓** (39)		
Fibrilin	**↑** (42)	**↑** (39)		**↓** (39)		
Fibronectin	**↑** (50)	**↑** (39)	**↑** (36)	**↓** (39)	**↓** (36)	Fibronectin is important for zebrafish cardaic regeneration (48)
Hyaluronic acid	**↑** (42)		**↑** (51)		**↓** (37)	Hyaluronic acid inhibition blocks zebrafish cardaic regeneration (43, 49)
Laminin		**↓** (39)	**↓** (36)	**↑** (39)	**↑** (36)	
Periostin	**↑** (50)	**↑** (39)	**↑** (52)	**↓** (39)	**↓** (37)	Periostin invovled in neonatal, and not adult, cardiac regeneration (47, 53–55)
Perlecan		**↑** (39)		**↓** (39)		
Thrombospondin			**↑** (37)		**↓** (37)	
Versican			**↑** (37)		**↓** (37)	

↑, *denotes high expression of selected ECM component*.

↓, *denotes low expression of selected ECM component*.

As with the discovery of HA and fibronectin, recent work has also identified agrin, a member of the proteoglycan family, to be highly enriched in the ECM of fetal/neonatal murine hearts and not in the hearts of adult mice (46). Interestingly, purified recombinant agrin had a pro-proliferative effect on cardiomyocytes cultured *in vitro*. When mice exposed to MI were administered with an intramyocardial injection of agrin, a significant improvement in cardiac function was observed with a reduction in overall myocardia injury. To further explore the cardiac regenerative potential of agrin, recombinant agrin was administered to porcine ischemia-reperfusion (IR) model (45). As in the murine setting, agrin improved cardiac function and reduced cardiac remodeling following IR injury. Although this field of research is still in its infancy, these observations do suggest that an increased understanding of the ECM may prove invaluable to unlock the intrinsic regenerative potential of the adult myocardial tissue.

## Discussion

### ECM Driven Cardiac Regeneration: Where Do We Go From Here?

Although significant advances have been made in the field of cardiovascular medicine, diseases such as myocardial infarction (MI) and ischemic heart disease, ultimately resulting in heart failure, remain amongst the leading causes of fatalities in the developed world. The mortality of these cardiovascular diseases is in no small part due to the irreversible loss of vessels and cardiomyocytes resulting in a decreased cardiac functionality. Current therapeutic strategies are aimed at alleviating the symptoms rather than repairing the loss of functioning cardiac tissue. Thus, researchers have turned to the field of regenerative medicine to identify novel strategies aimed at regenerating and/or repairing the number of functioning cardiac muscle cells within the failing heart.

Based on the current state-of-the-art, it is becoming increasingly evident that the utilization of stem-cell based approaches to achieve regeneration of the adult mammalian myocardium may not be the silver bullet scientists originally hoped for. Over the past decades several breakthroughs have been made in stem-cell biology that have enabled researchers to investigate numerous cell sources for cardiac regenerative purposes, and although some of these studies have demonstrated improvements in cardiac function in the translational setting, actual regeneration of the myocardium has yet to be demonstrated. This suggest that many of the cell sources for cardiac regeneration may hold a paracrine effect on the myocardium post-injury. Therefore, it has become evident that novel approaches have to be explored to attain true regeneration of the damaged myocardium.

**Figure 1:**
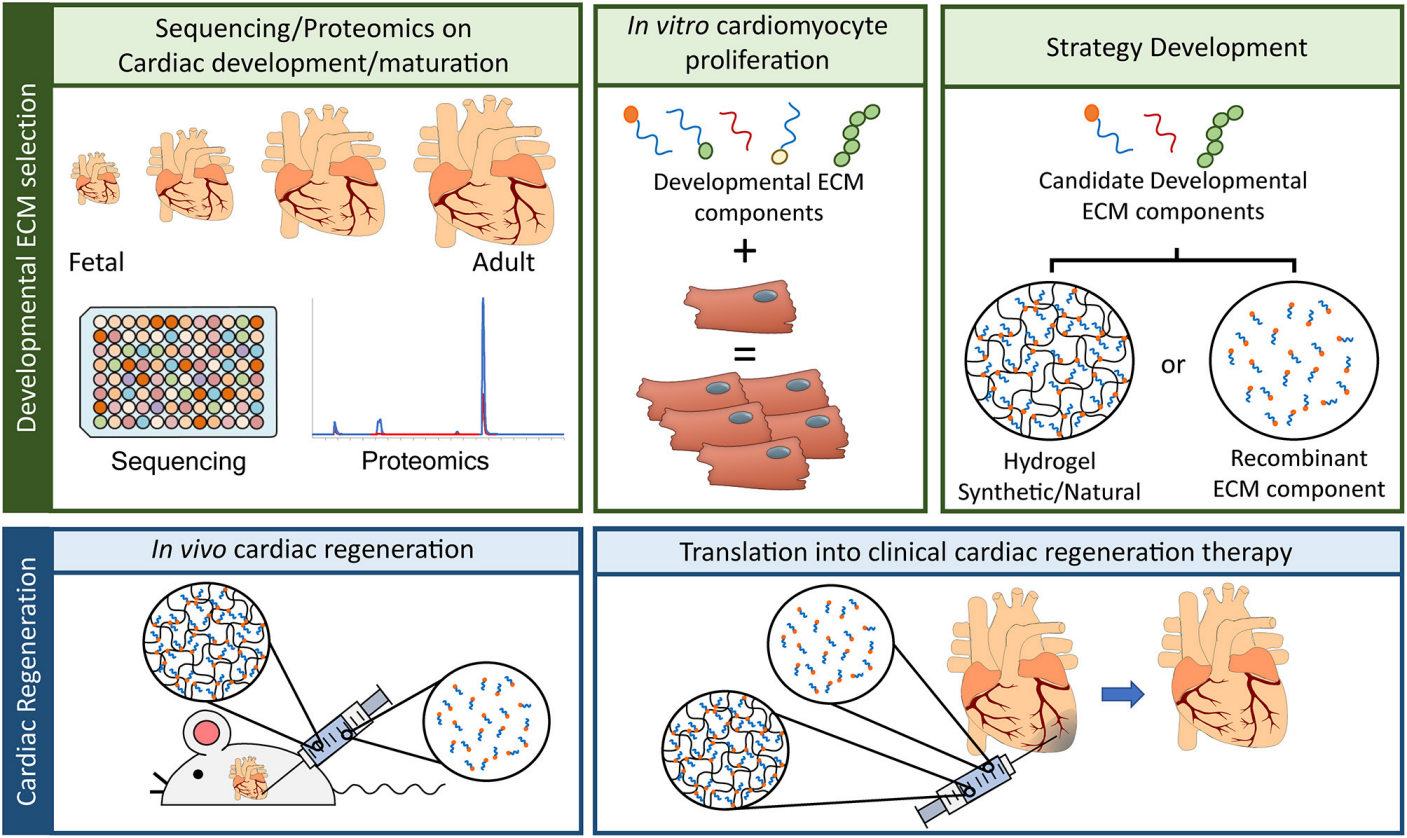
Advancement in ECM based cardiac regenerative research. To advance the field of cardiac regeneration it is essential to take advantage of the innate regenerative capacity of the fetal mammalian heart. “Developmental ECM selection” will require: (1) the utilization of advancements in “-omics” techniques, such as RNA sequencing and proteomics, to identify key ECM components present in the fetal/developing heart, (2) developmental ECM components will need to be screened *in vitro* for their capacity to influence cardiac regeneration, a key component of which is the induction of cardiomyocyte proliferation, and (3) the delivery of candidate developmental ECM components will have to be determined, either by means of a naturally produced ECM hydrogel, a synthetically produced ECM hydrogel, or by direct administration of the recombinant ECM component (TOP panel, from left to right). Having selected the most suitable ECM component for cardiac regeneration and the delivery system, these will have to be tested for their *in vivo* capacity to induce cardiac regeneration. The first step will revolve around animal model experiments, either in a rodent model (as depicted) or in a large mammal model (i.e., pig) for heart failure. Here the developed hydrogel harboring the ECM of interest or the recombinant ECM solution will be administered to the animals to determine their efficacy in inducing cardiac regeneration and improving overall cardiac function. The final step, and most crucial, will be to translated this ECM based therapeutic strategy into the clinic.

This is where unraveling the regenerative capacities of the zebrafish and/or neonatal mammalian heart may come in to play. Specifically when looking at the ECM, since several studies have demonstrated that the ECM of zebrafish and neonatal mammalian hearts possess an innate regenerative potential when applied to injured adult hearts. The undisputable potential the ECM has to regenerate the damaged myocardium as also lead to the development of an injectable hydrogel derived from porcine decellularized myocardial ECM (VentriGel), which has recently also been tested in the clinical setting (57). This study tested the efficacy and safety of the treatment, nevertheless patients did demonstrate an overall improvement following VentriGel treatment. It must be note that this therapeutic strategy, although highly innovative, does possess the drawback of being derived from porcine hearts and one can therefore not exclude any interspecies complications that may arise from this. A more suitable approach would be to develop a therapeutic strategy, a hydrogel-based one, which is synthetic and encompasses only the ECM components required to attain innate cardiac regeneration. This idea is further strengthened by recent studies which characterized HA and agrin, components of the ECM, as possessing strong cardiac regenerative potentials. Therefore, future studies should aim at improving our knowledge of the fetal/neonatal ECM and thereby unraveling the regenerative potential of this ECM to be harnessed for the development of novel therapeutic strategies of HF patients (Figure 1).

To push the field of cardiac regenerative medicine forward, several hurdles will have to be overcome before we can develop therapeutic strategies to be implemented in a clinical setting. Firstly, we must understand the “why” neonatal mammalian or zebrafish ECM is able to induce adult cardiac regeneration. To this end advances in omics-based approaches, including proteomics and RNA-sequencing, will enable scientist to fully characterize the composition of these early ECM's and identify which components are no longer present in the adult myocardium (58). Next, we must determine “how” these candidate ECM components are able to induce repair/regeneration of the injured myocardium (*in vitro* and *in vivo*). Finally, and the most crucial step will be the development of these ECM components into a synthetic hydrogel to facilitate the move into clinical application. Advances in the field of synthetic biomaterial have demonstrated the potential for the generation of such synthetic ECM hydrogels (59, 60). Although still in their infancy, the move toward a synthetic ECM hydrogel will facilitate the clinical usability of such a therapeutic strategy. A recent study has demonstrated the potential of this by developing a degradable elastin-like polypeptide hydrogel, which mimics the ECM (61). Intramyocardial injection of this hydrogel in a sheep model for myocardial infarction lead to a reduction in fibrosis, increased angiogenesis, improved cardiac function and cardiomyocyte integrity in the border zone of the infarcts (61). Taken together, these steps will not only help our understanding of cardiac regeneration as a whole, but will also lead to an improved outcome for patients suffering from HF.

### ECM Driven Cardiac Regeneration: Limitations and Cavities

One of the greatest obstacles that has to be overcome is our current knowledge gap related to the demonstrated role the ECM, in particular the neonatal ECM, plays in cardiac regeneration (29, 30, 32–35). Understanding the composition of the neonatal ECM will give a great impulse in the field of cardiac regeneration. However, scientists working on studying the role of neonatal ECM components for regenerative purposes must keep in mind the various functions and effects ECM components have. A prime example of this was the discovery of periostin, an ECM-associated proteins, which like Agrin was found to be highly expressed in the neonatal mammalian heart and barely detectable in the adult heart (52). Periostin was found to induce cardiomyocyte cell cycle re- following MI, resulting in improved cardiac function (47, 53). However, in the adult murine heart knocking-out or overexpressing periostin was found to have no effect on cardiomyocyte proliferation (54). But, reducing the expression of periostin in cardiac fibroblasts did result in a reduction in adverse cardiac remodeling in a murine MI model (55). This demonstrates that not all fetal ECM components will have the same effect in the neonatal heart as in the adult heart, and scientists must be critical when unraveling the regenerative potential of the fetal/neonatal ECM.

## Author Contributions

AvdP and CB contributed to planning, writing, and critical reading of this manuscript. Both authors contributed to the article and approved the submitted version.

## Conflict of Interest

The authors declare that the research was conducted in the absence of any commercial or financial relationships that could be construed as a potential conflict of interest.
